# Variation in competition performance, number of races, and age: Long-term athlete development in elite female swimmers

**DOI:** 10.1371/journal.pone.0242442

**Published:** 2020-11-18

**Authors:** Dennis-Peter Born, Ishbel Lomax, Michael Romann

**Affiliations:** 1 Department for Elite Sport, Swiss Federal Institute of Sport Magglingen, Magglingen, Switzerland; 2 Swiss Swimming Federation, Bern, Switzerland; 3 College of Life and Environmental Sciences, University of Exeter, Exeter, United Kingdom; São Paulo State University (UNESP), BRAZIL

## Abstract

While talent development and the contributing factors to success are hardly discussed among the experts in the field, the aim of the study was to investigate annual variation in competition performance (AVCP), number of races per year, and age, as potential success factors for international swimming competitions. Data from 40’277 long-course races, performed by all individual female starters (n = 253) at the 2018 European Swimming Championships (2018EC) for all 10 years prior to these championships, were analyzed. Relationships between 2018EC ranking and potential success factors, i.e., AVCP, number of races per year, and age, were determined using Pearson’s correlation coefficient and multiple linear regression analysis. While AVCP was not related to ranking, higher ranked swimmers at the 2018EC swam more races during each of the ten years prior to the championships (*P* < 0.001). Additionally, older athletes were more successful (*r* = -0.42, *P* < 0.001). The regression model explained highly significant proportions (*P* < 0.001) and 43%, 34%, 35%, 49% of total variance in the 2018EC ranking for 50m, 100m, 200m, and 400m races, respectively. As number of races per year (*β* = -0.29 –-0.40) had a significant effect on ranking of 50-400m races, and age (*β* = -0.40 –-0.61) showed a significant effect on ranking over all race distances, number of races per year and age may serve as success factors for international swimming competitions. The larger number of races swum by higher ranked female swimmers may have aided long-term athlete development regarding technical, physiological, and mental skill acquisitions. As older athletes were more successful, female swimmers under the age of peak performance, who did not reach semi-finals or finals, may increase their chances of success in following championships with increased experience.

## Introduction

During recent decades, improved performance of elite female athletes has narrowed the gap in performance to 8–12% compared to male athletes [1]. In swimming, depending on the discipline, the gender gap in performance is as low as 5% [1, 2]. With the improved swimming performance and minimal gap between top swimmers [3], marginal differences distinguish between first and second place, such as at the 2018 European Long-Course Swimming Championships (2018EC), where the women’s Freestyle final was won by only 0.01s and a 0.04% performance difference. As female swimmers reach elite performance three years earlier than males, potential success factors may be found throughout the early athlete development process at junior level [4]. As swimming has been performed in 50m pools for decades and performance is hardly influenced by environmental factors, such success factors throughout the long-term development of young swimmers may be investigated based on competition performances and race results [4]. Water temperature must be 25 to 28°C and with a current ≤ 1.25 m per minute. Pool length has a +0.01m tolerance and electronical time keeping is obligated in official competitions [5]. These standardized conditions facilitate comparison of race results across various competitions, venues, and seasons.

Previous studies analyzed race results and performance variations throughout the Olympic cycle [6], within the preparatory phase for the seasonal main event [7, 8], and between and within single competitions [7, 9]. It has been shown that performance became more stable leading up to the Olympics [6] and that better athletes demonstrated less variation in performance between competitions [9, 10]. Although gender differences in performance were not significant, there was a trend towards a larger performance variation in female swimmers [9, 10]. Furthermore, swimmers were more consistent within their main swimming stroke across various race distances rather than within a given race distance across various swimming strokes [9]. The authors concluded that swimmers are stroke rather than distance specific, and associated these findings with the high technical demand of swimming. However, it remains unclear whether performance variation differs across the various swimming strokes and race distances [7–10]. However, in most studies significant effects were missing and only marginal differences were reported. As conclusions were drawn based on mixed samples of males and females, analysis of performance variation across the years prior to international championships with particular focus on female swimmers could provide important insights into long-term athlete development.

Previous studies mostly evaluated performance variation in competitions of major importance [7, 9]. It can be assumed that athletes were well prepared and specifically tapered for these races. However, multiple competitions are swum in the stepwise progression towards peak performance [11, 12] and preparatory races may add an essential component to long-term athlete development based on psychological and physiological considerations [13–17]. Specific skill acquisition and experience, that comes with participation in high-level international competitions, seem to improve success chances in later stages of an athlete’s career [18, 19]. However, little is known about competition experience in regard to number of races per year and how performance may vary across all races swum throughout the season.

Undoubtedly, talent development and success in elite athletes is based on multiple factors, i.e., genetics, economics, and support system [20]. However, there is a heated debate on the effect of age and accumulated practice over time on elite athletes’ performance and level of expertise [20–22]. Indeed, age of peak performance in female athletes has increased throughout recent decades along with the improved peak performances [1, 23]. In particular, endurance performance seems to benefit from the accumulated hours of training, due to the progressive development of aerobic capacity and movement economy, needed for long race distances [24]. Hence, age of peak performance in endurance sports, i.e. running, cycling, and triathlon, increased with longer race distances [25, 26]. However, in female pool swimmers the opposite trend has been shown. Age of freestyle peak performance in female swimmers continuously decreased over distance, from 26.1±4.0 yrs for 50m to 21.9±1.5 yrs for 800m races [27]. With these conflicting findings, the question arises whether age contributes to success and is related to ranking of female swimmers at important international competitions, i.e. the 2018EC.

Improved swimming performance [3], minimal gap in performance between top swimmers [2], and plateauing of female elite performance during recent years has been reported [1]. As only 39% of study participants were women in three major sport sciences journals [28], previous research emphasized the need for comprehensive performance evaluations and identification of success factors specific to elite female athletes that are not solely derived from males [28, 29]. Therefore, the aim of this study is to analyze potential success factors for international swimming competitions, i.e., annual variation in competition performance (AVCP), number of races per year, and age, with particular focus on female swimmers throughout the 10-years prior to the 2018EC. It is hypothesized that athletes ranked higher at the 2018EC (1) will show higher consistency in performance with lower AVCP, (2) will compete in a greater number of races per year, and (3) are older than their lower ranked counterparts.

## Materials and methods

### Participants

From all female participants competing in individual events at the 2018EC in Glasgow (n = 253, age 21.7 ± 4.0yrs) all long-course races were analyzed for each of the 10 years preceding the championships. The study was approved by the institutional review board of the Swiss Federal Institute of Sport Magglingen and is in accordance with the Declaration of Helsinki (Reg.-Nr. 088_LSP_250919).

### Experimental design

All data were retrieved from the publicly accessible database ‘swimrankings.net’. In total, 40’277 races were analyzed using Person’s correlation coefficient and multiple linear regression analysis to determine the relationship between the 2018EC ranking and AVCP, number of races per year, as well as age. For the dependent variable, ranking was determined by the total number of swimmers permitted to compete in the various events and varied between swimming strokes and race distances (refer to Table 3).

### Data analysis

The number of races and AVCP were determined for each swimmer and for each of the 10 years prior to the 2018EC. The coefficient of variance was calculated with the standard deviation divided by the mean swimming times in that particular year and event to derive AVCP. A minimum of two races per year in the same event were necessary to calculate AVCP. Both, AVCP and number of races per year were analyzed for each swimming stroke, i.e., Butterfly, Backstroke, Breaststroke, Freestyle, and Individual medley, and each race distance (50m to 1’500m). Age in the year of the 2018EC was added to the regression model as the third independent variable.

Previous studies showed that swimming performance improved by 3–4% within five seasons leading up to the Olympic games [6]. Additionally, junior athletes showed a performance variation of 0.9–1.9% between two major competitions [9]. For the present study, races were analyzed with no information on tapering strategies, pacing, or potential health issues, such as neuro-muscular injuries or immune function. For instance, due to an injury or illness, a swimmer may have given up the race when lying far behind showing a race result far off the actual personal best. Therefore, the outlier analysis was customized in line with to the common mathematical approach, i.e. more than two times of the standard deviation [30], and variations > 4% within one year were defined as outliers. From a total of 4’400 coefficients of variance, only 59 coefficients were > 4% and had to be excluded from the analysis.

### Statistical analyses

Normal distribution was confirmed using standardized residuals and predicted values based on recommendations for large sample sizes by Field [30]. Data were evenly distributed in a random pattern around zero in the scatterplot, regression standardized residuals showed a Gaussian distribution in the histogram, and data displayed a diagonal straight line in the normal probability plot [30]. For the initial descriptive approach, AVCP, number of races per year, and age were correlated with the 2018EC ranking for all ten years preceding the championships (2018–2009) using Pearson’s correlation coefficient. Coefficients of < 0.3, 0.3–0.5, 0.5–0.7, and > 0.7 were classified as small, medium, large, and very large, respectively [31]. Means ± standard deviations were reported for all ten years prior to the 2018EC and compared with a two-way analysis of variance (ANOVA). If a main effect was found, *post-hoc* comparison was performed according to Bonferroni. Swimming strokes were compared using pooled data of the 50m, 100m, and 200m events. Race distances were compared within freestyle events only, as it is the only swim style swum for the full range of races distances (50m, 100m, 200m, 400m, 800m, and 1’500m). For the mechanistic analysis, multiple linear regression analysis was used to predict ranking at the 2018EC as the depended variable, based on AVCP, number of races per year, and age. Mean values of the last two years (2018–2017) prior to the 2018EC were used for the regression model. An alpha level of 0.05 was applied to determine statistical significance and Cohen’s *f* ^2^ calculated to determine size of the effects in the regression model [32]. The *f* ^2^ effect sizes of 0.02, 0.15, and 0.35 were classified as small, medium, and large, respectively [32]. Data were collected and prepared with Microsoft Excel 2016 (Microsoft Corporation, Redmond, WA, USA) for the subsequent statistical analysis with SPSS software package for Windows (Version 25.0, IBM Corporation, Armonk, NY, USA).

## Results

### Descriptive analysis

The AVCP (Table 1) and number of races per year (Table 2) were correlated with ranking at the 2018EC and the correlation coefficients with corresponding mean ± standard deviation are presented in Tables 1 and 2. The AVCP decreased throughout the ten years prior to the 2018EC for all swimming strokes (*P* < 0.001) and was lowest for Freestyle (*P* ≤ 0.04) but not different between Butterfly, Backstroke, Breaststroke, and Individual medley. Correlation analysis revealed no relationship between AVCP and 2018EC ranking.

**Table 1:** Pearson correlation (*r*) between annual variation in competition performance and ranking at the 2018 European Long-Course Swimming Championships with corresponding mean ± standard deviation in italic letters for all ten years prior to the championships.

	2018	2017	2016	2015	2014	2013	2012	2011	2010	2009
**All strokes and distances**	-0.09	-0.06	0.04	-0.11	-0.04	-0.03	-0.10	-0.06	-0.11	0.10
	*0*.*014±0*.*006*	*0*.*014±0*.*006*	*0*.*015±0*.*006*	*0*.*015±0*.*006*	*0*.*016±0*.*007*	*0*.*015±0*.*007*	*0*.*015±0*.*007*	*0*.*015±0*.*007*	*0*.*016±0*.*008*	*0*.*019±0*.*008*
**Butterfly**	-0.11	-0.19	-0.08	-0.20	-0.10	0.07	-0.24	-0.02	-0.36	-0.28
	*0*.*013±0*.*007*	*0*.*014±0*.*006*	*0*.*014±0*.*006*	*0*.*015±0*.*006*	*0*.*016±0*.*007*	*0*.*015±0*.*007*	*0*.*014±0*.*006*	*0*.*017±0*.*009*	*0*.*017±0*.*009*	*0*.*019±0*.*007*
**Backstroke**	-0.05	-0.02	0.06	-0.12	0.12	-0.04	-0.21	-0.06	-0.24	0.20
	*0*.*015±0*.*007*	*0*.*015±0*.*007*	*0*.*015±0*.*007*	*0*.*016±0*.*006*	*0*.*017±0*.*007*	*0*.*016±0*.*007*	*0*.*014±0*.*007*	*0*.*016±0*.*006*	*0*.*017±0*.*006*	*0*.*019±0*.*008*
**Breaststroke**	-0.04	0.08	0.06	-0.04	-0.02	-0.10	-0.06	-0.14	0.02	0.15
	*0*.*014±0*.*005*	*0*.*015±0*.*006*	*0*.*016±0*.*007*	*0*.*016±0*.*007*	*0*.*017±0*.*007*	*0*.*016±0*.*007*	*0*.*016±0*.*008*	*0*.*015±0*.*008*	*0*.*017±0*.*008*	*0*.*020±0*.*009*
**Freestyle**	-0.11	-0.20	0.12	-0.13	-0.06	-0.01	0.05	0.06	0.04	0.28
	*0*.*012±0*.*005*	*0*.*013±0*.*005*	*0*.*014±0*.*005*	*0*.*014±0*.*006*	*0*.*013±0*.*006*	*0*.*014±0*.*006*	*0*.*014±0*.*007*	*0*.*013±0*.*006*	*0*.*015±0*.*007*	*0*.*017±0*.*009*
**Individual medley**	-0.10	-0.27	-0.31	0.04	-0.06	-0.50	0.05	0.09	-0.23	0.05
	*0*.*014±0*.*004*	*0*.*018±0*.*006*	*0*.*017±0*.*006*	*0*.*019±0*.*006*	*0*.*017±0*.*004*	*0*.*018±0*.*008*	*0*.*016±0*.*007*	*0*.*017±0*.*006*	*0*.*016±0*.*007*	*0*.*022±0*.*007*
**50m**	0.00	-0.36	0.12	-0.23	-0.12	-0.15	0.16	-0.07	0.04	0.34
	*0*.*012±0*.*005*	*0*.*014±0*.*005*	*0*.*013±0*.*005*	*0*.*013±0*.*005*	*0*.*012±0*.*005*	*0*.*013±0*.*005*	*0*.*014±0*.*006*	*0*.*013±0*.*006*	*0*.*014±0*.*006*	*0*.*017±0*.*009*
**100m**	-0.20	-0.17	0.11	-0.15	0.03	0.07	-0.13	0.08	-0.14	0.26
	*0*.*013±0*.*006*	*0*.*012±0*.*005*	*0*.*014±0*.*006*	*0*.*014±0*.*006*	*0*.*013±0*.*006*	*0*.*013±0*.*005*	*0*.*013±0*.*006*	*0*.*013±0*.*007*	*0*.*015±0*.*007*	*0*.*016±0*.*009*
**200m**	-0.09	-0.05	0.15	0.01	-0.07	0.10	0.11	0.17	0.29	0.33
	*0*.*013±0*.*004*	*0*.*013±0*.*005*	*0*.*014±0*.*005*	*0*.*014±0*.*006*	*0*.*014±0*.*006*	*0*.*016±0*.*008*	*0*.*015±0*.*008*	*0*.*014±0*.*007*	*0*.*016±0*.*009*	*0*.*019±0*.*008*
**400m**	-0.21	-0.07	-0.02	0.09	0.31	0.11	0.45	0.24	-0.15	0.25
	*0*.*014±0*.*007*	*0*.*015±0*.*005*	*0*.*015±0*.*005*	*0*.*016±0*.*007*	*0*.*019±0*.*009*	*0*.*016±0*.*007*	*0*.*017±0*.*006*	*0*.*017±0*.*008*	*0*.*017±0*.*008*	*0*.*020±0*.*008*
**800m**	-0.28	-0.01	0.17	-0.20	0.22	0.63	-0.16	-0.65	-0.15	-0.36
	*0*.*012±0*.*003*	*0*.*011±0*.*005*	*0*.*014±0*.*005*	*0*.*013±0*.*006*	*0*.*013±0*.*005*	*0*.*013±0*.*005*	*0*.*017±0*.*010*	*0*.*015±0*.*006*	*0*.*018±0*.*010*	*0*.*018±0*.*006*
**1500m**	0.05	-0.46	0.59	0.63	-0.60	0.56	†	-0.71	†	-0.61
	*0*.*011±0*.*005*	*0*.*010±0*.*006*	*0*.*011±0*.*005*	*0*.*011±0*.*004*	*0*.*014±0*.*005*	*0*.*007±0*.*005*	*0*.*019±0*.*017*	*0*.*014±0*.*001*	*0*.*009±0*.*003*	*0*.*020±0*.*007*

† Number of observations < 3.

**Table 2:** Pearson correlation (*r*) between number of races per year and ranking at the 2018 European Long-Course Swimming Championships with corresponding statistical significance (*p*) in italic letters for all ten years prior to the championships.

	2018	2017	2016	2015	2014	2013	2012	2011	2010	2009
**All strokes and distances**	-0.22 ***	-0.28 ***	-0.29 ***	-0.32 ***	-0.25 ***	-0.23 ***	-0.26 ***	-0.21 ***	-0.28 ***	-0.25 ***
	*10*.*9±5*.*0*	*10*.*2±4*.*9*	*9*.*7±4*.*5*	*10*.*2±5*.*7*	*9*.*4±5*.*2*	*8*.*6±4*.*9*	*7*.*7±4*.*4*	*7*.*9±4*.*6*	*7*.*6±4*.*5*	*7*.*3±4*.*4*
**Butterfly**	-0.37 ***	-0.43 ***	-0.44 ***	-0.29 **	-0.29 *	-0.30 *	-0.35 **	-0.31 *	-0.36 *	-0.34 *
	*11*.*7±5*.*6*	*10*.*9±5*.*2*	*9*.*5±4*.*9*	*10*.*4±5*.*6*	*9*.*5±4*.*9*	*8*.*6±4*.*6*	*7*.*7±4*.*2*	*7*.*7±4*.*3*	*7*.*0±3*.*8*	*7*.*4±3*.*7*
**Backstroke**	-0.43 ***	-0.41 ***	-0.44 ***	-0.43 ***	-0.42 ***	-0.14	-0.21	-0.03	-0.01	-0.07
	*10*.*8±4*.*9*	*10*.*2±4*.*7*	*9*.*7±4*.*5*	*10*.*6±6*.*1*	*10*.*1±5*.*4*	*9*.*1±5*.*1*	*8*.*1±4*.*1*	*8*.*5±4*.*4*	*8*.*4±4*.*4*	*7*.*1±4*.*7*
**Breaststroke**	-0.29 **	-0.31 **	-0.21 *	-0.22 *	-0.28 **	-0.23 *	-0.14	-0.30 **	-0.25 *	-0.36 **
	*11*.*8±5*.*2*	*10*.*9±4*.*7*	*10*.*0±4*.*1*	*10*.*9±5*.*3*	*10*.*7±5*.*8*	*9*.*1±5*.*0*	*7*.*9±4*.*3*	*8*.*3±4*.*8*	*8*.*1±5*.*1*	*7*.*3±4*.*4*
**Freestyle**	-0.32 ***	-0.46 ***	-0.41 ***	-0.44 ***	-0.27 **	-0.35 ***	-0.42 ***	-0.29 **	-0.46 ***	-0.19
	*11*.*3±4*.*9*	*10*.*5±4*.*9*	*10*.*0±4*.*6*	*9*.*6±5*.*2*	*8*.*9±5*.*2*	*8*.*1±4*.*7*	*7*.*4±4*.*5*	*7*.*5±4*.*6*	*6*.*7±3*.*9*	*6*.*5±4*.*2*
**Individual medley**	-0.31	-0.20	-0.50 *	-0.57 **	-0.65 **	-0.44	-0.42	-0.38	-0.38	-0.59
	*10*.*6±3*.*9*	*10*.*6±5*.*1*	*11*.*0±5*.*6*	*13*.*3±9*.*1*	*8*.*3±5*.*0*	*8*.*1±5*.*1*	*6*.*5±3*.*0*	*7*.*3±4*.*3*	*7*.*3±4*.*4*	*8*.*1±5*.*1*
**50m**	-0.35 *	-0.51 ***	-0.53 ***	-0.54 ***	-0.35 *	-0.46 **	-0.38 *	-0.206	-0.42 *	0.19
	*10*.*9±4*.*7*	*10*.*7±5*.*6*	*9*.*8±4*.*5*	*9*.*8±5*.*2*	*9*.*1±4*.*3*	*8*.*8±4*.*9*	*7*.*6±4*.*6*	*7*.*7±4*.*3*	*6*.*3±3*.*6*	*6*.*0±3*.*3*
**100m**	-0.31 *	-0.53 ***	-0.36 *	-0.40 **	-0.15	-0.27	-0.39 *	-0.23	-0.49 *	-0.14
	*11*.*3±5*.*5*	*10*.*1±4*.*9*	*10*.*2±5*.*0*	*9*.*2±5*.*5*	*9*.*1±5*.*6*	*8*.*1±4*.*5*	*8*.*2±4*.*5*	*8*.*4±4*.*9*	*7*.*4±4*.*3*	*6*.*7±4*.*2*
**200m**	-0.30 *	-0.27	-0.34 *	-0.38 *	-0.34 *	-0.35 *	-0.62 ***	-0.53 *	-0.60 *	-0.58 *
	*11*.*8±4*.*5*	*10*.*7±4*.*0*	*10*.*0±4*.*6*	*9*.*7±4*.*9*	*8*.*4±5*.*9*	*7*.*4±4*.*6*	*6*.*1±4*.*3*	*6*.*1±4*.*5*	*6*.*1±3*.*9*	*6*.*8±5*.*5*
**400m**	-0.12	-0.32	-0.26	-0.57 **	-0.38	-0.58 **	-0.26	-0.30	-0.85 **	-0.39
	*9*.*5±4*.*2*	*9*.*9±5*.*1*	*9*.*8±4*.*6*	*8*.*8±4*.*8*	*8*.*2±4*.*2*	*8*.*6±6*.*1*	*9*.*2±7*.*4*	*8*.*8±7*.*2*	*11*.*1±7*.*1*	*13*.*8±4*.*8*
**800m**	0.05	-0.09	0.12	-0.32	-0.20	-0.21	0.36	-0.06	-0.01	-0.55
	*7*.*2±2*.*8*	*6*.*4±2*.*9*	*7*.*1±2*.*8*	*6*.*3±2*.*4*	*5*.*9±2*.*8*	*7*.*1±3*.*7*	*6*.*3±3*.*9*	*6*.*4±3*.*2*	*8*.*2±4*.*8*	*8*.*6±3*.*7*
**1500m**	-0.36	-0.17	0.00	-0.11	-0.33	0.78	†	-0.14	†	-0.14
	*4*.*8±1*.*9*	*4*.*6±1*.*7*	*3*.*8±1*.*5*	*3*.*3±1*.*2*	*4*.*8±0*.*8*	*3*.*2±1*.*3*	*3*.*0±1*.*4*	*3*.*3±1*.*5*	*5*.*5±2*.*1*	*4*.*3±1*.*5*

* *P* < 0.05.

** *P* < 0.01.

*** *P* < 0.001.

† Number of observations < 3.

**Fig 1:**
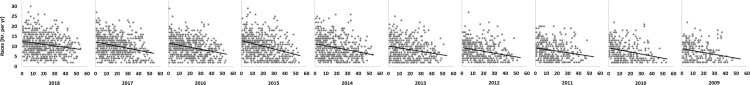
Correlation matrixes for number of races per year in regard to ranking at the 2018 European Long-Course Swimming Championships. Data points represent individual values for each participant and year across all swimming strokes and race distances.

The number of races per year increased throughout the ten years prior to the 2018EC for Butterfly (*P* < 0.001), Backstroke (*P* < 0.001), Breaststroke (*P* < 0.001), and Freestyle (*P* < 0.001), but not Individual medley. Regarding race distance, there was an interaction effect (*P* < 0.001) that showed an increasing number of races per year for 50m (*P* = 0.002), 100m (*P* = 0.003), and 200m races (*P* = 0.009). Across all swimming strokes and race distances, swimmers who ranked higher at the 2018EC participated in significantly more races for all ten years prior to the championships (*P* < 0.001). Correlation analysis revealed small to medium effects for most of the ten years prior to the championships for Butterfly (*r* = -0.29 –-0.44), Breaststroke (*r* = -0.21 –-0.36), and Freestyle (*r* = -0.27 –-0.46). These effects were less clear but evident for most of the five years prior to the championships for Backstroke (*r* = -0.41 –-0.44) and the Individual medley (*r* = -0.50 –-0.65). Refer to Table 2. Regarding race distance, higher ranking correlated with significantly more races for most of the years prior to the 2018EC with medium to large effects for 50m (*r* = -0.35 –-0.54), 100m (*r* = -0.31 –-0.53), and 200m races (*r* = -0.30 –-0.62). However, based on correlation analysis, association between ranking and race participation was unclear for 400m, 800m, and 1500m races (Fig 1).

**Fig 2:**
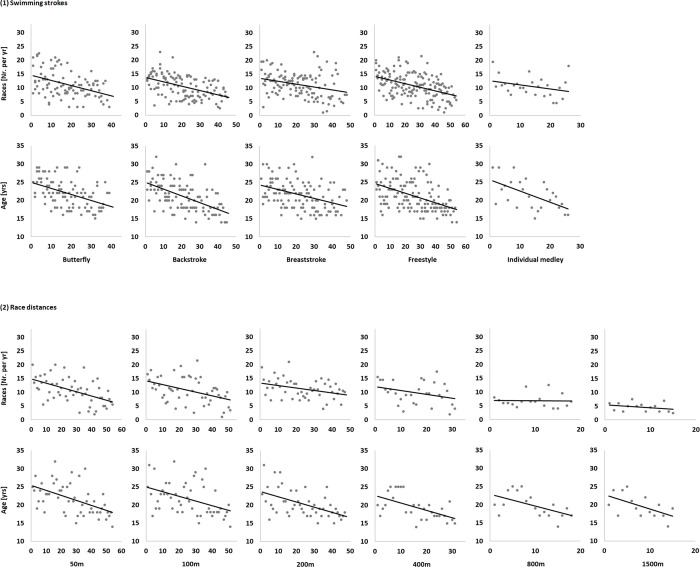
Correlation matrixes for number for races per year (races) and age in regard to ranking at the 2018 European Long-Course Swimming Championships. Data points represent individual values for each participant across the various swimming strokes and race distances.

Older athletes were generally more successful at the 2018EC across all swimming strokes and race distances (*r* = -0.42, *P* < 0.001; Fig 2). This age effect was apparent across all swimming strokes, i.e. Butterfly (*r* = -0.47, *P* < 0.001), Backstroke (*r* = -0.58, *P* < 0.001), Breaststroke (*r* = -0.40, *P* < 0.001), Freestyle (*r* = -0.46, *P* < 0.001), and Individual medley (*r* = -0.57, *P* = 0.002), and all race distances, i.e. 50m (*r* = -0.52, *P* < 0.001), 100m (*r* = -0.43, *P* = 0.002), 200m (*r* = -0.52, *P* < 0.001), 400m (*r* = -0.56, *P* = 0.001), 800m (*r* = -0.54, *P* = 0.020), and 1500m races (*r* = -0.56, *P* = 0.030).

### Mechanistic analysis

The regression model explained 22% (*P* < 0.001) of total variance of the 2018EC ranking with a medium effect size (*f* ^2^ = 0.28) for pooled data of all swimming strokes and race distances (Table 3). While the 2018EC ranking remained unaffected by AVCP, there was a highly significant effect for age and number of races, where higher ranked swimmers were older and participated in more races per year. This pattern was evident across all swimming strokes except for Individual medley, for which only age showed a significant effect on ranking. Regarding race distance, the regression model explained a highly significant proportion (*P* < 0.001) of the rankings with large effect sizes for 50m (43%, *f* ^2^ = 0.75), 100m (34%, *f* ^2^ = 0.52), 200m (35%, *f* ^2^ = 0.54), and 400m races (49%, *f* ^2^ = 0.96), with number of races per year and age having a significant effect on the dependent variable. While 800m races remained unexplained (*P* = 0.107), the regression model explained 50% (*P* = 0.049, *f* ^2^ = 1.00) of variance in the ranking of 1500m races with a significant age effect. None of the race distances were affected by AVCP.

**Table 3:** Multiple linear regression analysis for annual variation in competition performance (AVCP), number of races per year (races), and age. Mean values of the last two years prior to the 2018EC were used for the regression model.

	Regression model					Regression coefficients			
	Entries	*R* square	*f* square	*F* value	*P* value		Beta	*T* value	*P* value
**All strokes and distances**	589	0.22	0.28	*F*_(3,585)_ = 54	*P* < 0.001	AVCP	-0.04	*T* = -1	*P* = 0.310
						Races	-0.19	*T* = -5	*P* < 0.001
						Age	-0.39	*T* = -10	*P* < 0.001
**Butterfly**	95	0.29	0.41	*F*_(3,91)_ = 13	*P* < 0.001	AVCP	-0.01	*T* = 0	*P* = 0.891
						Races	-0.29	*T* = -3	*P* = 0.003
						Age	-0.37	*T* = -4	*P* < 0.001
**Backstroke**	116	0.46	0.85	*F*_(3,112)_ = 31	*P* < 0.001	AVCP	0.00	*T* = 0	*P* = 0.952
						Races	-0.36	*T* = -5	*P* < 0.001
						Age	-0.49	*T* = -7	*P* < 0.001
**Breaststroke**	119	0.25	0.33	*F*_(3,115)_ = 13	*P* < 0.001	AVCP	0.01	*T* = 0	*P* = 0.865
						Races	-0.29	*T* = -4	*P* < 0.001
						Age	-0.41	*T* = -5	*P* < 0.001
**Freestyle**	149	0.36	0.56	*F*_(3,145)_ = 27	*P* < 0.001	AVCP	-0.10	*T* = -2	*P* = 0.134
						Races	-0.36	*T* = -5	*P* < 0.001
						Age	-0.41	*T* = -6	*P* < 0.001
**Individual medley**	26	0.43	0.75	*F*_(3,22)_ = 6	*P* = 0.005	AVCP	-0.24	*T* = -1	*P* = 0.164
						Races	-0.19	*T* = -1	*P* = 0.278
						Age	-0.55	*T* = -3	*P* = 0.003
**50m**	52	0.43	0.75	*F*_(3,48)_ = 12	*P* < 0.001	AVCP	-0.04	*T* = 0	*P* = 0.758
						Races	-0.40	*T* = -3	*P* = 0.002
						Age	-0.43	*T* = -4	*P* < 0.001
**100m**	50	0.34	0.52	*F*_(3,46)_ = 8	*P* < 0.001	AVCP	-0.15	*T* = -1	*P* = 0.231
						Races	-0.36	*T* = -3	*P* = 0.005
						Age	-0.40	*T* = -3	*P* = 0.002
**200m**	47	0.35	0.54	*F*_(3,43)_ = 8	*P* < 0.001	AVCP	-0.05	*T* = 0	*P* = 0.675
						Races	-0.29	*T* = -2	*P* = 0.027
						Age	-0.47	*T* = -4	*P* < 0.001
**400m**	30	0.49	0.96	*F*_(3,26)_ = 8	*P* < 0.001	AVCP	-0.12	*T* = -1	*P* = 0.422
						Races	-0.38	*T* = -3	*P* = 0.014
						Age	-0.61	*T* = -4	*P* < 0.001
**800m**	18	0.34	0.52	*F*_(3,14)_ = 2	*P* = 0.107	AVCP	-0.20	*T* = -1	*P* = 0.384
						Races	-0.12	*T* = -1	*P* = 0.590
						Age	-0.58	*T* = -3	*P* = 0.020
**1500m**	15	0.50	1.00	*F*_(3,11)_ = 4	*P* = 0.049	AVCP	-0.10	*T* = 0	*P* = 0.662
						Races	-0.39	*T* = -2	*P* = 0.112
						Age	-0.62	*T* = -3	*P* = 0.016

## Discussion

The aim of this study was to analyze potential success factors for international swimming competitions. Correlation analysis showed that swimmers with a better 2018EC ranking were older and swam significantly more races in each of the 10 years leading up to the championships. The regression model predicted success chances and explained a highly significant proportion (*P* < 0.001) of 43%, 34%, 35%, and 49% of total variance in the 2018EC ranking for 50m, 100m, 200m, and 400m races, respectively, with number of races per year and age having a significant effect on the dependent variable. Ranking of 1500m races was explained by 50% in the regression model and affected by age only. While AVCP had no effect on 2018EC ranking, number of races per year for race distances ≤ 400m and age could serve as success factors for international swimming competitions, as they had a large effect on the dependent variable.

In previous studies, variability in competition performance decreased during the preparatory phase leading up to the seasonal main event [6, 8]. While better athletes generally showed a more stable performance [9], lower variability was found in short compared to long race distances [7]. These studies evaluated important events in the competition schedule, i.e., Pan Pacific Championships, Olympic Trials, Olympic Games, and National Championships, assuming that swimmers particularly prepared for these races [7, 9]. However, the present study included all races swum throughout the year, showing no difference in the AVCP between higher, i.e. finalists, and lower ranked swimmers, i.e. slowest swimmers in the heats, at the 2018EC. Additionally, swimmers with higher 2018EC ranking swam more races in each of the 10 years prior to the championships. Not all of these races can be swum fully prepared and with a complete 2–4 week taper period without accepting a substantial decline in training time over the course of the season [12]. Preparatory races of minor importance, for which athletes were not fully tapered and prepared for, may have caused results below the swimmers’ current personal best times. This could explain the difference in current findings compared to previously reported lower performance variation for better athletes [9] and the peaking phase [6, 8]. Additionally, conclusions drawn from mixed samples of males and females may not reflect performance variation in elite female swimmers.

The positive association between number of races per year and 2018EC ranking could be related to acquisition of important physiological, technical, and mental skills in preparatory races [13–17]. Therefore, race participation should always be integrated in a meaningful training process, rather than simply adding extra stress to young swimmers. As participation in competitions requires time and energy, i.e. travelling [33] athletes may benefit from swimming more races in the competitions they are participating in, rather than increasing the number of competitions. Probably due to the high technical demand of swimming [34], physiological capacity and mental skills can be more readily transferred over various distances, compared to technical skills, which are more difficult to adapt between strokes [9]. Therefore, swimmers are trained for a specific swimming stroke rather than race distance [9]. In preparatory races, this may allow swimmers to apply their main stroke to various distances and benefit technically from the race specific velocities [13]. Still, swimmers may increase physiological benefits from competing in various strokes over their main event’s race distance, as swimming races provide a very intense and effective form of training [14, 15].

Although such training races will most probably result in swim times below personal bests, a larger number of races could improve mental skill acquisition, i.e., dealing with defeat, coping with expectations, and developing resilience [17, 35, 36]. If athletes develop a task-oriented and growth mindset, unprepared races can be accepted as a challenge. However, athletes with a fixed and achievement-oriented mindset are more likely to avoid the possible failure associated with unprepared participation in races of minor importance, thereby neglecting further learning opportunities [37, 38]. With the right assessment of possible underperformance and failure in untapered training races, participating in a large number of races early on in their career could help athletes to build mental success factors, i.e., task-orientation, competition routine, and self-confidence, rather than fear and anxiety, for the benefit of long-term athlete development [16, 37].

Athletes with a higher 2018EC ranking were older over all swimming strokes and race distances, which is in line with previous studies that presented the concept of deliberate practice [22]. It is believed that a certain amount of practice, i.e., 10’000 hrs, within a given time span, i.e., 10 yrs, is required to achieve expert level. This theory is hardly discussed among experts in the field [20, 21, 39–43] and talent development most probably involves multiple factors [44]. Still, the high technical demand and specifics of in-water locomotion [9, 34] may favour volume based training approaches and accumulated practice over time to achieve elite performance [45]. With the usual talent pathway, children start competitive swimming at the age of 8–10 [46]. Due to earlier biological maturation [47], female swimmers reach a competitive level for elite performance three year earlier than males, which is as early as by the age of 18 yrs [4]. Depending on the race distance, performance peaks by the age of 21.9 to 26.1 yrs [27]. Therefore, Ericsson’s 10-year rule of deliberate practice seems to serve as rule of thumb for the talent pathway of male, as well female swimmers [22].

Although the present study hints towards an accumulated effect of race experience over time, i.e. more races per year were related to 2018EC success, the question arises whether this is the best way to achieve world-class performance. In contrast to the deliberate practice theory, previous research showed that later specialization and high variation in training and competition at a young age may help sustain motivation for high-level training and competition, develop important motor tasks, prevent overuse injuries, and eventually improve performance at senior level [41, 43, 48]. In particular, psychological skills, i.e., dealing with defeat, coping with expectations, and developing resilience [17, 35, 36] can be developed in any kind of competition. In the context of swimming, in preparatory races athletes may compete in their main stroke across various race distances and technically benefit from the race specific velocities [13]. For a physiological oriented conditioning, swimmers may compete in various strokes in their main event’s race distance [14, 15]. Later specialization in the development process of young swimmers or even on-land activities may help them reach the next level in swimming performance [41–43, 48].

Although age of peak performance has increased throughout the recent decades for females [23], age of peak performance in swimming is lower than in other endurance and explosive sports [23]. Additionally, age of peak performance decreases with increasing race distance, showing lower age for long-distance (21.9±1.5 yrs) compared to short-distance (26.1±4.0 yrs) pool swimmers [27]. As aerobic capacity and movement economy is built up over years [24], age of peak performance generally increases with increasing race distance in endurance sports [25, 26]. However, as drop-out in swimming is often due to motivational and mental reasons [49], age and window of peak performance may be substantially effected by psychological, rather than physiological factors [25, 26, 49]. Based on the present findings, where older swimmers were more successful at the 2018EC, swimmers under the age of peak performance who did not reach semi-finals or finals are encouraged to focus on mental skills and keep up the motivation for high-level training and competition, as chances of success may increase with the next championship participation.

### Methodological considerations

In the present study, competition results were extracted with no information on the swimmers’ personal background. Therefore, a large data set across all participants of the championships were used to account for potential interferences, such as training process, potential health issues, or involvement in other sports across 10 years. Future studies should investigate the underlying mechanisms and reasons for the higher number of competitions in higher ranked swimmers using surveys and questionnaires. As the dependent variable was determined by the total number of athletes permitted to compete in the various championship events, ranking varied from 15 to 54 swimmers. As such, results of longer events with a lower number of participants, i.e. 800m and 1500m Freestyle, should be interpreted with care.

The aim of the study was to evaluate novel factors that are related to success of female swimmers at international competitions. As medium correlations were found between ranking and number of races per year, future studies should quantify the effect of race participation within the multi-dimensional model of talent development, including many other factors, i.e. social background, training modalities, and genetics [20, 50]. With the reduced gender difference in performance throughout recent years [1, 2], lower age of elite and peak performance in female swimmers [4, 27], and previously emphasized need for comprehensive performance evaluations and identification of success factors specific to elite female athletes [28, 29], for the present study, all female swimmers competing in single events at the 2018EC were included. As potential gender effects may exist, future studies should investigate AVCP, number of races per year, and age in male swimmers. Additionally, potential differences between total number of races and number of competitions, training history, and skill transfer between swimming strokes are important aspects that need further consideration.

## Conclusions

Previous studies showed that performance became more stable towards the Olympics [6] and that better athletes’ performance varied less between competitions of particular importance [9, 10]. However, in the present study 2018EC ranking was unaffected by AVCP when analyzing all races swum throughout the year. Although, the present study showed that higher ranked female swimmers competed in more races per year, number of competitions should not simply be increased. Instead of just adding extra stress to young swimmers, races should be implemented purposefully in a reasonable training process to aid technical, physiological, and mental skill acquisition. Future studies should verify these findings, with a detailed analysis of the underlying mechanism. Despite the debate on the effect of age and accumulated practice over time among experts in the field of talent development [20–22], in this study older female athletes were more successful at the 2018EC. While swimmers may not improve their performance simply by aging, the present study should encourage swimmers under the age of peak performance, who did not reach semi-finals or finals at international competitions, to focus on mental skills and motivation to continue the hard work of high-level training and competition. Experience and chances of success may increase in following championship participations.

## Supporting information

S1 Data (XLSX)
